# Analysis of the Key Factors Affecting the Capability and Optimization for Magnetostrictive Iron-Gallium Alloy Ambient Vibration Harvesters

**DOI:** 10.3390/s20020401

**Published:** 2020-01-10

**Authors:** Huifang Liu, Chen Cong, Chongdong Cao, Qiang Zhao

**Affiliations:** 1 School of Mechanical Engineering, Shenyang University of Technology, Shenyang 110780, China; congchenc3c@163.com (C.C.); caochongdong@126.com (C.C.); zq15169213405@126.com (Q.Z.); 2 Department of Architecture and Civil Engineering, City University of Hong Kong, Kowloon, Hong Kong SAR 999077, China

**Keywords:** harvester, iron-gallium alloy, magnetostrictive, structural configuration, bias condition, energy harvesting capability

## Abstract

The basic phenomena of a cantilever energy harvesting device based on iron-gallium alloy magnetostrictive material for low frequency were systematically studied. The results highlighted how the physical parameters, geometric structure and bias conditions affected the vibration harvesting capacity through a thorough experimental aimed at enhancing the vibration energy harvesting capacity through an optimal design. How the performance is affected by the configuration of the multi-layers composite beam, material and dimensions of the elastic layer, arrangement position and number of bias magnets, the matching load resistance and other important design parameters was studied in depth. For the first time, it was clearly confirmed that the magnetic field of bias magnets and electromagnetic vibration shaker have almost no effect on the measurement of the voltage induced from the harvester. A harvesting power RMS up to 13.3 mW and power density RMS up to 3.7 mW/cm^3^/g was observed from the optimized prototype. Correspondingly, the DC output power and power density after the two-stage signal processing circuit were up to 5.2 mW and 1.45 mW/cm^3^/g, respectively. The prototype successfully powered multiple red light emitting diode lamps connected in a sinusoidal shape and multiple red digital display tubes, which verified the vibration harvesting capability or electricity-generating capability of the harvester prototype and the effectiveness of the signal converter.

## 1. Introduction

Harvesting ambient energy is an effective way to achieve sustainable green energy for portable and wireless electronic devices, especially those that can operate for long periods of time without human intervention. Traditionally, batteries are the primary source of power for such devices. However, the billions of batteries produced each year are unsustainable, their disposal poses environmental problems, and their limited service life poses a challenge to the long-term/autonomous operation of the devices. The rapidly growing market for solid-state electronics is leading the development of ultra-low-power devices and self-powered devices [1], such as wearable electronic devices [2], biosensors [3], IoT and remote sensors [4,5]. Accordingly, the world is very interested in reducing carbon dioxide emissions and developing more renewable energy technologies to generate electricity. Among them, vibration power generation relying on wind flutter [6,7], bridges [8,9] and automobiles [10] is one of the fastest growing energy sources in the world, and is actually a non-polluting renewable energy source. In the last decade, many scholars have been actively developing a range of energy harvesters that can convert tiny amounts of mechanical energy, especially harvesting energy from low frequency mechanical vibrations to generate usable electricity to power standalone electronic systems. The electromagnetic power generation method which operates on the principle of Faraday’s law of electromagnetic induction is one of the most pivotal vibration harvesting methods. It can provide sufficient power from strong magnetic fields and the power generation capacity depends on the relative movement between the coil and magnet. Ahmad et al. [11] designed a flow-based electromagnetic energy harvester using a microplanar coil for IoT sensor applications. The prototype volume was 291.4 cm^3^ and experimentation indicated that it was able to generate a 686 μW of maximum power at an operating flow rate of 12 L/min at the optimal matching impedance. Liu et al. [12] presented a non-resonant rotational electromagnetic energy harvester for harvesting irregular human motions. The prototype had a weight of 2 kg and was able to provide a maximum power of 10.4 mW Not a power unit. However, the generated voltage depends on the product of the vibration frequency and the size (coil’s interlinkage area), which results to the electromagnetic energy conversion systems having relatively large mass and bulk, and consequently, miniaturization of the electromagnetic generator in order to integrate with some electronic devices for convenient use is difficult. Moreover, under weak magnetic fields with low frequency, the power generated from an electromagnetic device is disappointingly small.

Compared with traditional passive energy harvesters, smart materials, including piezoelectric and magnetostrictive materials, can exhibit higher energy conversion characteristics at lower frequencies, because they harvest and convert energy through the inherent properties of smart materials rather than utilizing relative motion between components. This characteristic can help reduce the system mass and bulk and be beneficial for integration with application devices. Smart materials have been proven to harvest vibration energy from the environment, for example, from mechanical operations [13], wind [14], and human motion [15]. Piezoelectric vibration energy harvesting is currently the most widely studied and most popular smart material used in vibration energy harvesting, because it can provide a practical way to scavenge energy from the environment to power nanodevices and nanosystems, and can also be used as novel self-powered sensing devices [16]. Liu [17] at Shanghai Jiao Tong University proposed a piezoelectric Y-type wind energy harvesting method. Kim et al. [18] developed a highly flexible P(VDF-TrFE) film-based energy harvesting device on a PDMS substrate. Dong and Wen et al. [19] developed a piezoelectric energy harvester which was employed to utilize the bending of the lead of a cardiac pacemaker or defibrillator for generating electrical energy. Gradually, piezoelectric-based energy harvesting methods are combined with triboelectric or electromagnetic technology to form hybrid energy harvesting devices [20,21]. However, such piezoelectric materials have issues such as depolarization, charge leakage [22], difficulty in achieving the impedance matching to maximize the output power [23] and other disadvantages [24].

In magnetostrictive materials (MSMs) which are a new type of smart material, mechanical stress causes changes in magnetization and this property is known as the Villari effect [25]. The idea of vibration energy harvesting based on MSM uses the change of magnetic flux density due to the oscillation of a structure (usually a cantilever beam) and generates a voltage throughout a pick-up coil. At lower frequencies, a MSM energy harvester produces higher power density than other types of energy harvesters which is a prominent advantage since the fabrication of low frequency energy harvesting devices is a challenging dilemma. MSM energy harvester possesses another obvious advantage in that it is inductive, which can power a low impedance at the fundamental frequency of common vibration sources. In addition, in MSM harvesters, limitations such as brittleness, depolarization, aging and the need for an external charge or voltage source, which are usual in other methods, vanish [26]. Terfenol-D is a typical commercial MSM with a chemical composition of TbDyFe, whose high energy conversion coefficient is up to 70%, while that of Ni-based alloys is only 16% and that of the commonly used PZT material is only 0–60%. However, the defects such as high brittleness, high stiffness and low cycle life, result in it being unsuitable for applications that require flexibility and long-term work. In recent years, MSM such as magnetostrictive ductile iron alloys (referred to as Galfenol) and glass fiber metal compounds (such as Metglas) have gradually emerged. Galfenol—iron-gallium alloy—is a new promising MSM. It can be easily machined, it is weldable, and has a tensile strength of approximately 500 MPa [27] and hence can be obtained in different shapes and sizes which might be useful for innovative devices, so it can be used for almost infinite vibration cycles, which can significantly improve the reliability of system operation. Vibration harvesters based on iron-gallium alloy appear to be able to provide better energy harvesting and conversion performance due to their physical properties, such as high energy density and mechanical properties similar to steel. In 2010, Yoo and Flatau [28] conducted an interesting early study to demonstrate the effective energy conversion of Galfenol. They presented a proof-of-concept prototype Galfenol-based energy harvester and obtained the result that the output voltage was proportional to the load-induced bending of the Galfenol element. Iron-gallium alloy-based cantilever harvesters have recently been developed. In 2011 and 2012, Ueno et al. [24,29] illustrated the performance of cantilever iron-gallium alloy harvesting devices with respect to their electromechanical layout. In [30], the dimensions of pick-up coils, beam thickness ratio and tip mass for base excitation harvester were parameterized by the finite element method. In [22,31], the performance of a Fe-Ga-based harvester has been investigated, in terms of output voltage, power and efficiency, as well as interface circuit assuming the load resistance and the impressed acceleration as a parameter. Cao [32] and Haynes [33] established the model and carried out experimentally test for the similar configurations. Apicella et al. studied the magneto-mechanical optimization issues of a magnetostrictive cantilever beam bonding with Al sheet for energy harvesting [34]. In 2017, Clemente et al. [35] presented an equivalent circuit which was a nonlinear three-port circuit, in which, the magneto-mechanical modeling was quite realistic and exploited nonlinear functions and the full coupling among the involved physical quantities of the employed magnetostrictive material. In 2019, Clemente et al. [36] presented a nonlinear equivalent circuit of a harvester based on multiple rods of Galfenol, which was able to predict the output voltage and harvested power for different loads and pickup coils connections. Compared to the piezoelectric cantilever harvesters dating back to the 1990s, further efforts are needed to optimize this technology by thoroughly understanding the underlying phenomena that occur throughout the harvester. It requires considerable effort to control all parameters that may affect its performance for the precise design of the cantilever vibration harvester.

The aim of this study is to provide an outline, based on a thorough experimental study, of the basic phenomena affecting the behavior of such iron-gallium alloy based cantilever harvesting systems and, in particular, how the physical and geometric structure, bias magnetic field conditions, and matching impedance affect the overall output performance, which lays a foundation for the later development of its practical application research. It studies in depth the basic problems of the cantilever iron-gallium alloy vibration energy harvester, especially the interaction between mechanical structure and experimental equipment, to ensure a device with sufficient repeatability. The influences of the electromagnetic coil of experimental equipment (shaker), magnetic lines of flux of the bias magnets on the induced voltage in pick-up coils are excluded. It clearly describes the basic design issues and outlines the design principles of the cantilever iron-gallium alloy vibration energy harvester. Furthermore, it carries out experiments of powering and continuously lighting multiple light emitting diode lamps and digital tubes by the harvester prototype, so as to further verify the sustainable electricity-generating capability of the designed iron-gallium alloy vibration harvester and the effectiveness of the proposed energy storage conversion method.

## 2. Structural Analysis and Prototype Development of Vibration Composite Beam

When we begin to develop the cantilever harvester based on iron-gallium, the first issue to be considered is the system geometry and its construction.

### 2.1. Structural Configuration of Vibration Composite Beam

**Figure 1:**
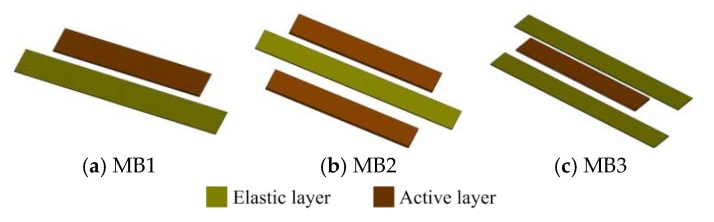
Exploded view of the multi-layers composite beam consisted of active layer and elastic layer. (**a**) MB1: one active layer bonded on the upper surface of elastic layer; (**b**) MB2: two active layers bonded on the both sides of one elastic layer; (**c**) MB3: one active layer sandwiched between two elastic layers.

**Figure 2:**
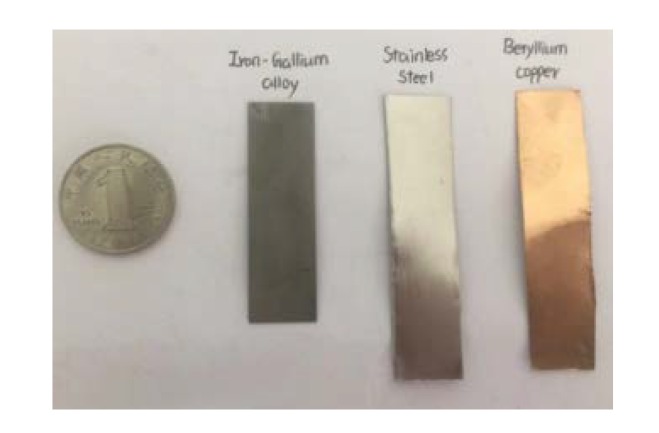
Photographs of active layer and elastic layer samples compared with a Chinese one yuan coin.

The whole structure of the harvesting device is a composite beam construction comprising MSM bonded on an elastic layer and wound by pick-up coils. In more detail, the apparatus should include an iron-gallium alloy active layer, and an elastic layer capable of at least improving the mechanical properties of the iron-gallium alloy material in terms of rigidity and strength, such as stainless steel, aluminum foil and beryllium copper, etc. Therefore, the first issue is to choose the optimal multi-layer structure that provides the best power conversion capacity without increasing the complexity of the layer-to-layer overlay. To this end, we have designed three multi-layer structure layouts, as shown in Figure 1. The first one (referred to as MB1) is by bonding an active layer to an elastic layer. The second one (referred to as MB2) is by bonding two active layers on the both sides of one elastic layer. Instead, the third one (referred to as MB3) is by bonding one active layer sandwiched between two elastic layers. Two types of materials (steel, beryllium copper) with the same dimensions are chosen as the elastic layer in this paper. In Figure 2, the involved active layer and elastic layer samples are shown. For all the composite beam prototypes, the elastic layer and active layer are bonded together utilizing a commercial 3MDP100 two-component epoxy adhesive, as well as curing the pick-up coils.

### 2.2. Development of Harvester Prototype

**Figure 3:**
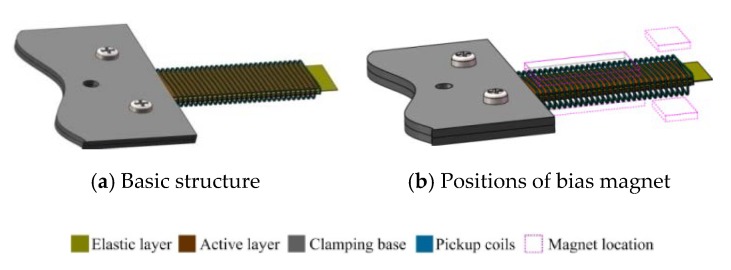
Structure of the harvester prototype.

The developed structure of the MSM iron-gallium alloy based cantilever harvester prototype is shown in Figure 3. A 40 mm × 15 mm × 0.5 mm polycrystalline iron-gallium alloy beam (as the active layer) manufactured by TdVib Ltd. (Kunshan, JiangSu Province, China) is bonded to the elastic layer. The gallium concentration of the as grown ingot is about 13% Ga, which is close to that of the starting polycrystalline alloy. The pick-up coils wire diameter is selected as 0.203 mm (AWG32) and the number of coil turns is 800, with 12.2 Ω resistance and 5.8 mH inductance (measured with configuration MB2). The elastic layer is made of stainless steel or beryllium copper with the same width and thickness as the iron-gallium layer, and the length is the same as or slightly longer than that of the iron-gallium active layer. Material and length selection of the elastic layer are analyzed in detail through experiments in Section 4.2. All multi-layer composite beam structures should be pre-magnetized through a magnetic field provided by a suitable set of permanent magnets. The possible positions at which the permanent magnets may be arranged include on the upper surface of the beam, at the lower surface of the beam, on the above (or below) of the beam with a certain distance, which are indicated by a pink dashed box in Figure 3b. NdFeB magnets are used in every case. From experiments, it find that the magnet configuration has a strong influence on the overall vibration harvesting performance of the prototype, and the detailed research will be elaborated in Section 4.3. A clamping base intended to clamp the tested multi-layers composite beam, the material of which is aluminum alloy, is also shown in Figure 3. The composite beam is clamped by the two upper and lower clamping bases through bolts. The base is fixed above the vibration shaker by stud, so that the composite beam is in the form of a cantilever. The distance between the composite beam and shaker is adjusted by stud to avoid the measurement being disturbed from the electromagnetic field in the shaker.

## 3. Experimental System Design, Experimental Conditions Analysis and Determination

**Figure 4:**
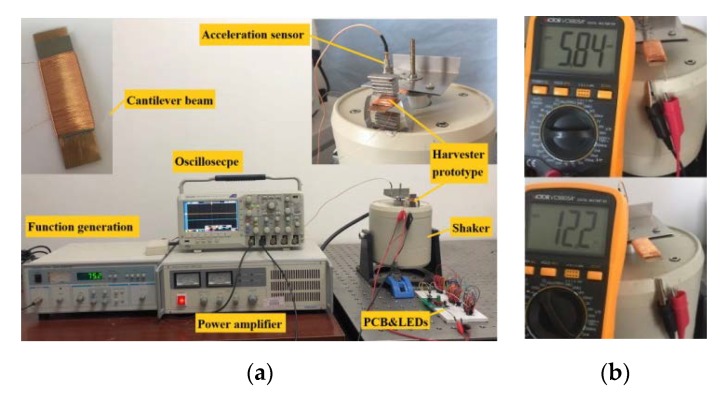
Experimental setup. (**a**) Photograph of actual experiment setup. Inset shows the fabricated prototype; (**b**) Photograph of measuring the impedance parameters of the pick-up coils.

A photograph of the whole experimental system is shown in Figure 4. The key experimental equipment is a JZK-20 electromagnetic vibration shaker (Sinocera, Yangzhou, China) that provides a mechanical energy source with controllable frequency and excitation amplitude. The vibration harvester prototype is connected to the output end of the shaker through the clamping base. The Sinocera YE1311 function signal generator can generate a small amplitude sinusoidal signal with any frequency, whose output signal is amplified by the Sinocera YE5837A power amplifier and used to drive the shaker. The vibration exciter applies vibration excitation to the vibration composite beam through the clamping base of the prototype, and the excitation form is acceleration. The Sinocera CA-YD-130 accelerometer (frequency response range is 2–5000 Hz, and sensitivity is 3 pC/g) is used to measure the acceleration of the composite beam. The displacement of the free end of the composite beam is measured by a non-contact laser displacement sensor. Tektronix DPO2014B digital phosphor oscilloscope is used to monitor and record the acceleration and output voltage of the prototype and the output signal from the interface circuit. The experimental tests are carried out at the frequency of 5–105 Hz and acceleration of 1–6 g.

### 3.1. Determination of the Height of Clamping Base

**Figure 5:**
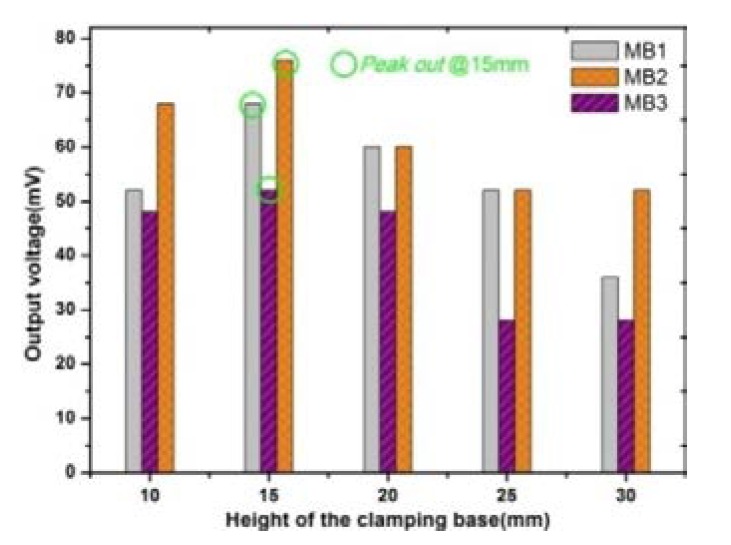
Height of the clamping base analysis: 5g acceleration, without bias magnet.

The distance between the output end of the vibration shaker and the composite beam (i.e., the height of the clamping base) may have an effect on the transmission efficiency of vibration. In order to quantify the optimal height, we have fabricated three types of composite beams with different arrangements according to Figure 1. Three types of composite beams, which have the same active layer and elastic layer, and the elastic layer is 10 mm longer than the active layer, are equipped with the same pick-up coils without a bias magnetic field. Their fundamental resonance frequencies are measured by Lissajous Figure method, which are 65, 75 and 154 Hz, respectively. At the fundamental resonance frequency, the output voltages of all the three composite beam prototypes are measured one by one and compared, where the clamping base height range from 10 mm to 30 mm. The result is shown in Figure 5. The composite beam equipped with a base of 15 mm height generates the largest voltage and the maximum value (amplitude) is about 76 mV. In this case, the vibration harvesting prototype can receive and harvest vibrations to the greatest extent. If the composite beam is equipped with a higher clamping base, its vibration harvesting and conversion capacity unexpectedly become weaker because of the increasing of system damping. Therefore, in the subsequent experiments, the height of the clamping base was fixed at 15 mm.

### 3.2. Analysis of the Influence of the Electromagnetic Coils in Vibration Shaker, Permanent Magnets on the Induced Voltage

**Figure 6:**
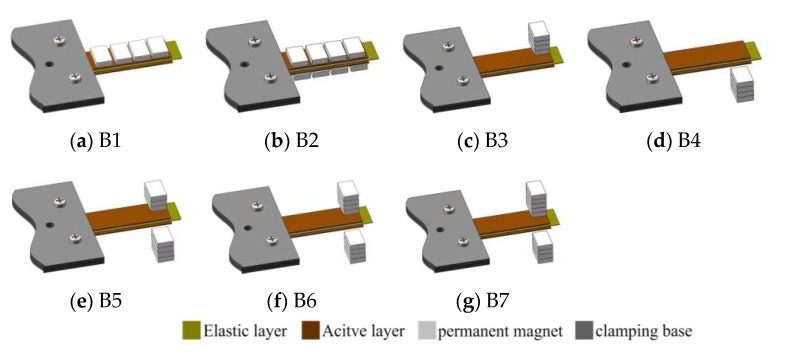
Position and layouts of the bias magnets in the harvester.

**Figure 7:**
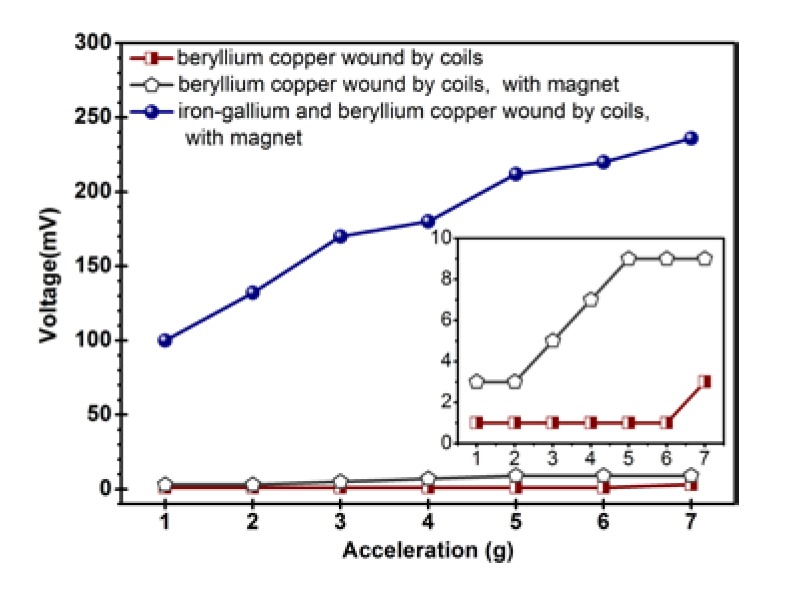
Open-circuit voltage measured on the harvesting device with and without iron-gallium alloy layers or magnets.

Although the composite beam is separated from the shaker surface by 15 mm through the clamping base, the bias magnetic field of the permanent magnet and the magnetic field generated by the electromagnetic coils in the vibration shaker may affect the measurement by introducing an unwanted voltage on the pick-up coils. In order to quantify these two undesired effects, we compared the behavior of the composite beam of MB1 and that of the same beryllium copper cantilever beam which has no iron-gallium layer and is equipped with the same coil, under the conditions of with or without permanent magnet configuration. Permanent magnets are arranged below the composite beam (see Figure 6d). The result is shown in Figure 7.

It is noted that, even if the acceleration reaches a high level (7 g, where 1 g = 9.8 m/s^2^), the voltage generated in the pick-up coils by the magnetic field of permanent magnet or the electromagnetic field of shaker is still less than 9 mV, which is much less than the 236 mV generated by the vibration energy harvester prototype. In addition, we also tried to configure the composite beam with 500 and 1500 turn pick-up coils, and the induced voltages generated by these two effects are still very small, even at such high accelerations. For example, the generated voltages are less than 5 mv and 10 mV at 7 g acceleration, respectively. Therefore, one can ignore approximately the influence of the permanent magnet magnetic field and the electromagnetic field in the vibration shaker on the experimental measurement results.

## 4. Comprehensive Experiments and Detailed Analysis

### 4.1. Analysis of the Structural Configuration of Multi-Layer Composite Beam

**Figure 8:**
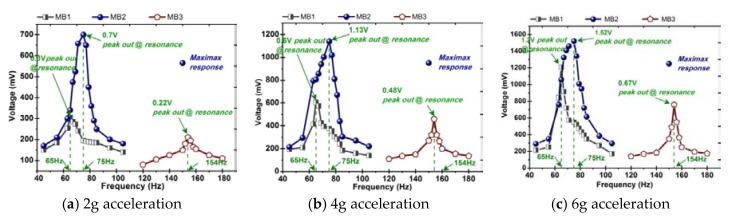
Comparison of voltage frequency-response at different acceleration performed with the beam in different configurations: MB1, MB2 and MB3. Magnets are arranged as B6, shown in Figure 6f.

In this section, we conduct a test and analysis for the composite beams with different structures shown in Figure 1. In order to obtain a reasonable results comparison, all the prototypes are with the same bias condition that eight permanent magnets are arranged in a vertically symmetrical manner, as shown in Figure 6f. In particular, a harmonic vibration is applied to the prototype in the frequency range (45–180 Hz) with a vibration acceleration amplitude of 2–6 g. The measured open-circuit voltage from the prototype is shown in Figure 8. The first set of test is performed on the composite beam with only one iron-gallium active layer (i.e., the arrangement of MB1 and MB3). In the former case, the maximum output voltage amplitude is about 1.2 V, and the corresponding fundamental resonance frequency is 65 Hz, at 6 g acceleration. In the latter case, the maximum output voltage is one order of magnitude lower, about 0.67 V, and the corresponding fundamental frequency is 154 Hz. The phenomenon of small output voltage for MB3 configuration is explained as follows. We observe the composite beam of MB3 configuration and find that its structure distributes symmetrically. The symmetrical structure causes the stress in the cross section to be symmetrically distributed with respect to the neutral plane which is the zero stress plane as the beam bent. In the configuration of MB3, the neutral plane is located in the middle of the composite beam, which coincides with central plane of the iron-gallium layer. Beam bending creates a tensile state above the plane and a compressed state below the plane (and vice versa). The magnetization produced by the Villari effect increases above the neutral axis, while decreases below the neutral axis, which results that the Villari effect is completely or at least partially offset on the whole. Thereby reduces the variation in the magnetic flux within the pick-up coils, and consequently, the induced voltage at the terminals of pick-up coils. The second set of test is performed on a composite beam (MB2) with two iron-gallium alloy active layers. As a result, the maximum output voltage is about 1.52 V and the fundamental frequency is 75 Hz. It is apparent that the prototype with two active layers has a stronger ability of harvesting and converting vibration into electric energy than that of with single layer. Moreover, the obtained voltage spectra curves show the asymmetry and shift of the resonant frequency from 65 Hz (MB1), 75 Hz (MB2) to 154 Hz (MB3) with different multi-layer configurations.

**Figure 9:**
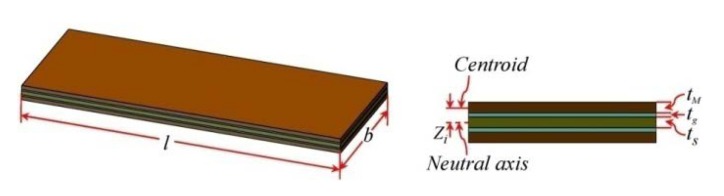
Geometry of the multi-layers composite beam with iron-gallium alloy layers.

Figure 9 shows a composite cantilever beam geometry with multiple active layers. The length and width of composite beams are expressed by *l* and *b*, respectively. It shows several iron-gallium alloy layers (brown, as annotated by *t*_M_) bonded on the elastic layer (olive-green, as annotated by *t*_s_) using adhesive layers (blue-green, as annotated by *t*_g_). The thickness of different layers *t*_s_, *t*_g_ and *t*_M_, as well as *Z_i_*, are shown in the figure as well. *Z_i_* is the distance from the centroid of the *i*_th_ active layer to the neutral axis.

Assuming *k* numbers of iron-gallium alloy layers are bonded on the elastic layers using the same number of adhesive layers, the effective distance from the centroid of the iron-gallium alloy layers to the neutral axis *h*_M_ can be expressed as [37]:

(1) $$h_{M}=\sum_{i=1}^{k}Z_{Mi}=\frac{kE_{s}t_{s}\left(t_{g}+t_{s}\right)+k^{2}\left(E_{s}t_{s}+E_{g}t_{g}\right)\left(t_{g}+t_{M}\right)}{2\left(E_{s}t_{s}+kE_{M}t_{M}+kE_{g}t_{g}\right)}$$

in which, *E* is the Young’s modulus, where subscripts *s*, *g* and *M* indicate the elastic, adhesive and iron-gallium alloy layer, respectively.

When *ω* = *ω*_n_, open-circuit voltage at the fundamental resonance frequency is [37]:

(2) $$V_{oc}=\frac{F_{0}Nd^{*}E_{M}A_{M}h_{M}\eta \lambda }{l\left(c_{m}+f_{c}\left(k\right)\right)\left(\sqrt{l^{2}/4+r_{coil}^{2}}+r_{coil}\right)}$$

where, *F*_0_ is the magnitude of the Laplace transform of the excitation force in the thickness direction of the beam, *N* is the number of turns of the pick-up coils, d* is a magneto-mechanical coefficient, *A*_M_ is the cross-sectional area of the iron-gallium layer which equals *bt*_M_, *c*_m_ is the mechanical damping, *r*_coil_ is the coil radius. *f*_c_(*k*) is the mechanical damping related to the number of active layers. *λ* and *η* are coefficients of mode shape functions associated with the first normal mode, and can be obtained by solving the mode shape function [38,39].

The induced voltage and output power increase as the active layer thickness *h*_M_. However, there are two factors that limit the number of active layers that can be bonded to the elastic layer. First, the natural frequency of the harvester increases as the active layer, which is undesirable in low frequency vibration energy harvesting applications, because it reduces the output power density of the entire system. Second, larger mechanical damping will be created by the adhesive layer. If the increase of *h*_M_ due to more active layers is higher than the corresponding increase of mechanical damping, the open-circuit voltage increases, and vice versa. In addition, in the manufacturer process of the prototype, there are more or less non-uniform in the adhesive layers, and other defects such as bubbles in the adhesive layers, which also reduce the induced voltage and the output power.

In summary, the above preliminary experiments outlines that performance of the vibration harvester has a significant dependence on the configuration of the composite beam, and the structure with multiple active layers is more suitable for obtaining high vibration harvesting and electrical energy conversion capacity. The research result is consistent with those reported in [34]. Also, there is an optimal number of active layers to maximize output voltage and power. The detailed optimization design of the active layer will be studied in detail in the future.

### 4.2. Analysis of the Material and Size of Elastic Layer

Many studies have investigated MSM-based cantilevered vibration energy harvesting devices, which consist of interconnected substructure beams (elastic layer and MSM layer). The elastic layer is fabricated from a material intended to improve the mechanical properties of the MSM in terms of rigidity and strength, such as stainless steel, beryllium copper, aluminum foil etc. The MSM vibration energy harvesting devices designed in [31,40] use aluminum as the elastic layer. The MSM vibration energy harvesting devices designed in [22,26,37] select stainless steel as the elastic layer. In [41,42], copper is selected as the elastic layer of the presented MSM vibration energy harvesting devices. However, the basis for determining these material of the elastic layers is not described in these references. In this section, the effects of elastic layer of non-magnetic stainless steel and beryllium copper on the energy harvesting performance are measured in detail through experiments. Here, we focus only on the mechanical properties of the elastic layer on the impact of harvester, so all the elastic layer materials are non-magnetic. In the future, we will further study the influence of elastic layer on the distribution of magnetic field.

**Figure 10:**
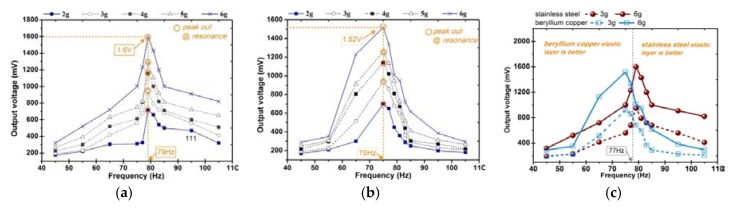
Voltage frequency-response at different acceleration performed with the beam with different elastic layer (multilayer configuration MB2, magnets configuration B6, elastic layer is 10 mm longer than active layer). (**a**) Stainless steel elastic layer, (**b**) Beryllium copper elastic layer, (**c**) Comparison of the output voltage of the beams with different elastic layers.

Experiments are carried out on the composite beam with MB2 configuration. A same bias conditions (B6, as shown in Figure 6f) are applied to all the prototypes. A harmonic vibration is applied in the frequency range (45–105 Hz) with an acceleration amplitude of 2–6 g, and meanwhile, the open-circuit voltage is measured. Figure 10 shows the result of the material of elastic layer on the vibration harvesting capacity. The composite beam with a stainless steel elastic layer has a fundamental natural frequency slightly larger than that of a composite beam with a beryllium copper elastic layer, and the generated voltage at resonance is also slightly larger. The maximum output voltage of stainless steel is 1.6 V and beryllium copper is 1.52 V. When the vibration frequency is less than 77 Hz, the generated voltage of the prototype with beryllium copper elastic layer is slightly larger than that of with stainless steel elastic layer; and vice versa. The results show that the material of the elastic layer has a certain influence on the output of vibration harvester, but it is not very significant. However, it has a relatively larger effect on the fundamental resonant frequency. In all the latter experiments, elastic layers are all fabricated by beryllium copper because of low resonant frequency considerations.

**Figure 11:**
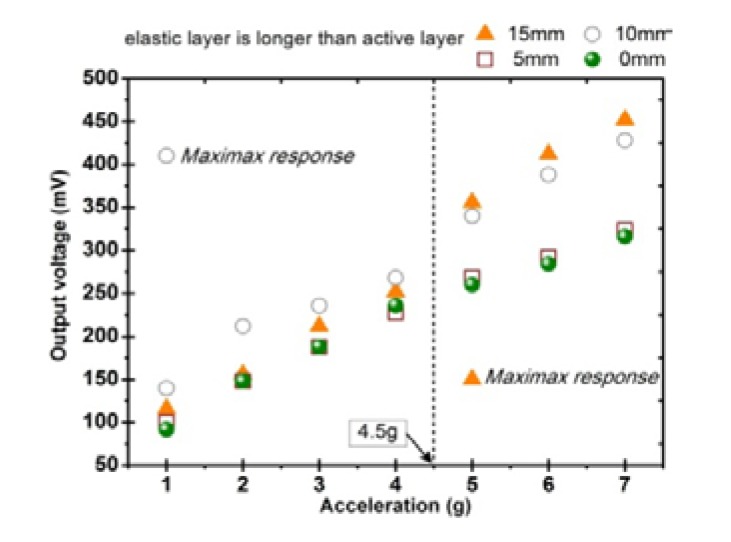
Voltage analysis at different acceleration performed with the beam with different length elastic layer.

Figure 11 shows the experimental results of the vibration harvesting capacity associated with the length of the elastic layer. The experiment is performed on a composite beam with MB1 configuration. It can be clearly observed that the composite beam with a beryllium copper elastic layer longer than the iron-gallium active layer is superior to the composite beam with a same length of elastic layer and active layer. This phenomenon can be explained to some extent by this: stress is the main factor for generating electrical energy; the longer the elastic layer, the greater the deflection of the iron-gallium layer, and correspondingly, the change in magnetic flux density and induced voltage increasing [43]. The composite beam with an elastic layer 10 mm longer than active layer has a vibration harvesting effect similar to that of 15 mm. When the vibration acceleration is lower than 4.5 g, the output voltage of the 10 mm layout is larger than that of 15 mm; and vice versa. Thus, the vibration harvesting capacities are similar in general. In the subsequent optimization of the bias magnetic field layout and other experiments, 10 mm layout is selected, which is from the consideration that the real ambient vibration acceleration may be not high.

### 4.3. Bias Magnetic Field Parametric Study

The function of the bias magnetic field on the initial state of magnetic domain and the Villari effect of MSM is similar to the effect of pre-tightening force on Joule effect. Many references [44,45] have mentioned that the bias magnetic field has an influence on the characteristics of MSM vibration harvesting devices. In most of the reports, the permanent magnet was just arranged directly in a fixed position on the harvester in a fixed form, however, it is not clear which arrangement can maximize the energy conversion capacity. Ueno and Yamada [24] proposed a micro energy-harvesting device, using two parallel beams of Galfenol. A permanent magnet with a yoke was attached to the structure to provide adequate bias flux for the beams. Yang and Tan et al. [22] proposed L-shaped MSM transducer to harvest energy from finger tapping. A neodymium magnet was attached near the free end of the L-shaped beam.

This section is devoted to analyzing the vibration harvesting performance of the composite cantilever beams with MB2 configuration under different bias magnetic field arrangements. The open-circuit output voltage of the harvesters without bias magnetic field or with bias magnetic field provided by permanent magnets are tested experimentally. Magnets are arranged in seven modes, as shown in Figure 6. The coercivity of every piece of magnet is 110 KA/m. Permanent magnets are close to the surface of cantilever beam with configuration B1 and B2. Permanent magnets are above or below the cantilever beam with configuration of B3, B4, B5, B6 and B7. Moreover, there is a certain distance between the magnet and cantilever beam.

**Figure 12:**
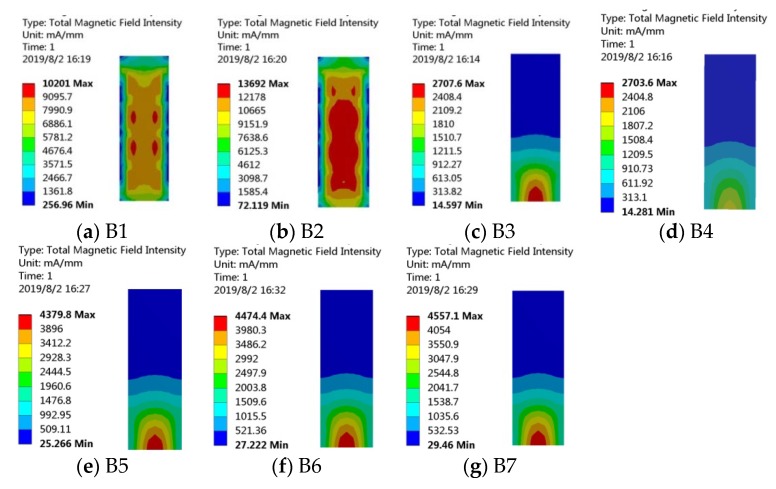
Magnetic field intensity distributions in iron-gallium layer of the harvesters with the above layouts of bias magnets.

Magnetic field intensity distributions are analyzed by the ANSYS Workbench method, and the results are shown in Figure 12. Magnetic field intensity in iron-gallium layers is distributed symmetrically along the width direction, which is able to ensure that the iron-gallium alloy is relatively fully utilized. It is noted that magnetic field intensity of B1 and B2 are much higher than that of others. Magnetic field intensity of B3 and B4 are the smallest. In the layouts of B5, B6 and B7, B6 is medium.

**Figure 13:**
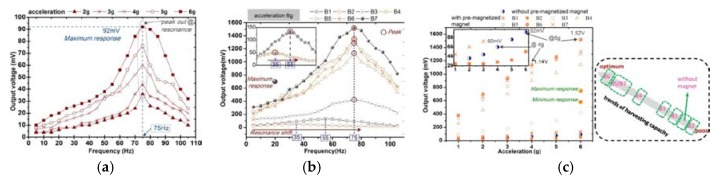
Open-circuit voltage performed with the beam with different bias magnet layouts. (**a**)without bias magnet, (**b**) with bias magnet, 6 g acceleration, (**c**) contrastive analysis at different bias magnet layouts at the fundamental resonance frequency.

Figure 13 shows the open-circuit voltage of the energy harvester with or without bias magnets. It can be seen that there is the effect of bias magnetic field in the iron-gallium layer, resulting in a higher output voltage. This is because bias magnetic field causes more domains to be stimulated and correspondingly, the magnetic domain will rotate widely and expand greatly once it is excited by an external mechanical stress, and thus strongly modify the magnetic properties of iron-gallium alloy due to the Villari effect. When the magnet is placed above the beam and in contact with the beam (layout B1), the output voltage is increased compared to the zero bias magnetic field. However, when the magnet is placed according to B2, the output voltage is smaller than the zero bias magnetic field. Meanwhile, the fundamental resonance frequencies of the energy harvesting system with magnet layouts of B1 and B2 are also increased; this is because the magnets add to the mass of the composite beam, which is equivalent to an additional weight of the cantilever beam. Although the magnets of B2 provides iron-gallium alloy with the most large bias magnetic field, the resonance frequency is too low, on the contrary, which results in a small induced voltage, which is attributed to the fact that the induced voltage largely depends on the resonance frequency. When the magnets are above or below the beam and are kept at a certain distance from the beam (layouts B3, B4, B5, B6, B7), the iron-gallium layer has a relatively high energy conversion capacity. Moreover, the fundamental resonance frequency does not increase. When the magnet is completely symmetrically distributed above and below the cantilever beam (layout B6), the iron-gallium layer senses a most suitable bias magnetic field, which has the strongest promoting effect on the energy conversion capacity. The maximum output voltage is increased from the very low level (92 mV) of none bias magnetic field to the maximum level (1.52 V) corresponding to the layout B6, which is increased by 16.5 times. As the bias magnetic field continues to increase (layout B7), the energy conversion capacity of iron-gallium layer is gradually reduced. On the one hand, this phenomenon can be explained by means of the magnetization curve of iron-gallium alloy MSM material which is shown as Figure 1 in reference [46]. It can be seen that when the bias magnetic field is zero, there is no flux change; next, as the bias magnetic field increases, the flux change gradually increases; then, as the magnetization saturation state is approached, the flux change gradually decreases to zero. This is also similar to the result described in reference [47], which is about the effect of bias magnetic field on the Villari effect. The energy conversion behaviors difference observed in the harvester with the symmetrical or asymmetrical arrangement of bias magnets can be explained by the content of literature [34]. It may be due to the combined effects of the non-uniform stress distribution in the heterostructure and the different bias magnetic field intensities at different positions in the active layer. A more detailed analysis for the effects of bias magnetic field requires detailed modeling for the entire harvesting system, which will be addressed in future research.

It can be obviously observed that the harvester with B6 bias magnet layout output the largest voltage. Layouts B4, B5, B5 and B7 are better than B1, B2 and B3. It can be seen that the maximum voltage reaches to 1.52 V (at 6 g acceleration), which also reaches 1.14 V even at moderate acceleration (4 g), which may be also larger than that reported in the literatures [44,48] about MSM vibration harvester to a certain extent. Although the voltage is smaller than that reported in the literature [34] (2 V at 4 g acceleration), the voltage density (voltage divided by the area of active layer) 1.9 V/cm^2^ is larger than that of [34] (1.73 V/cm^2^).

### 4.4. Analysis of the Optimal Matching Impedance and the Output Characteristics of Power

The amount of energy harvested from vibrations often remains very low, and hence the vibration energy harvesting system must operate at the optimal point for maximizing the power output. This section is devoted to clear the relationship between the output voltage and power and the connected external load in order to optimize the electrical output performance of the energy harvester system from the perspective of the loaded conditions.

The energy harvesting system acts as a voltage source during its operation powering the signal processing conversion circuit or the storage circuit, in turn a sensor or other independent electronic device. It is assuming that the energy harvesting system is a linear two-port circuit with independent power supply. The internal electric circuit of the harvester contains inductive component and works in a sinusoidal steady-state, therefore, we use Thevenin’s theorem and the the maximum power transfer theorem [49,50] in the complex field. The load matching condition that ensure that the external load can absorb the maximum power is that the load impedance and the equivalent impedance of the harvester’s electric network system are conjugated each other:

(3) $$Z_{L}=Z_{c}^{*}$$

or:

(4) $$X_{L}=X_{C}\;and\;R_{L}=R_{c}$$

*Z_L_* is the load impedance including the resistance *R*_L_ of the external load and the matching capacitance *C*. *Z*_c_ is equivalent impedance of the harvester including the resistance *R*_C_ and inductance *L*. *X_L_* is the internal inductive impedance. *X_C_* is the capacitance impedance of matching capacitor. *R_L_* is the resistance of external load. *R*_C_ and *L* are the resistance and inductance of pick-up coils respectively. *ω* is angular frequency:

(5) $$Z_{c}=R_{c}+jX_{L}=R_{c}+j\omega L$$

(6) $$Z_{L}=R_{L}+jX_{C}=R_{L}-j\frac{1}{\omega C}$$

**Figure 14:**
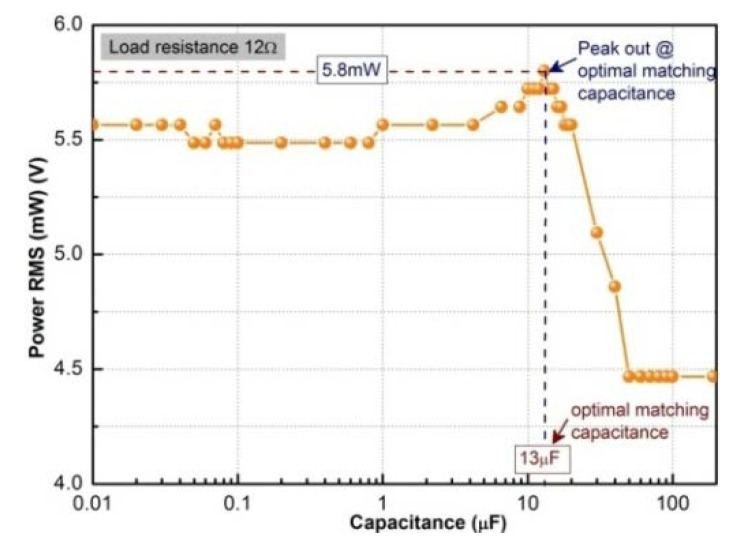
Matching capacitance optimization.

Based on the experimental results from the previous sections, an optimal harvester is designed. The elastic layer is selected as beryllium copper and is 10 mm longer than active layer. The multi-layers are configured in the form of MB2 and the bias magnet form is layout B6. In this experiment, we select pure resistors as the external load in series to the harvester. On the basis of the load matching conditions for maximum power shown in Equation (4), we choose a 12 Ω constant resistor in series with a tuning capacitor. The capacitance ranges from 0.01 to 190 μF. The first set of experiment is carried out under a harmonic vibration with 3 g acceleration and 75 Hz frequency. The relationship between the power absorbed by the resistor and the tuning capacitance is shown in Figure 14. The maximum output power is achieved when the capacitance is 13 μF.

**Figure 15:**
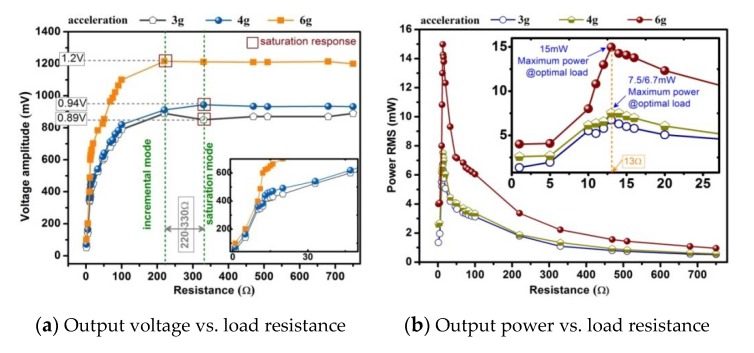
Matching resistance optimization (measured at 75 Hz).

**Figure 16:**
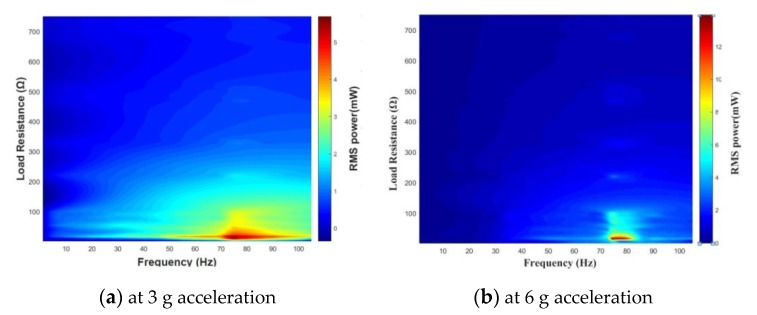
Three-dimensional pseudo-color diagram of power vs. load resistance and frequency, at 3g acceleration or 6g acceleration.

The second set of experiment is to determine the optimal resistance load. With the matching capacitance of 13 μF, the resistance load in series to the pick-up coils is gradually tuned in the range 1–750 Ω. Especially, we make a very detailed measurement near 12 Ω. The voltage across the resistor is shown in Figure 15a. As the load increases, the voltage gradually increases and approximately saturates at about 220–330 Ω until the voltage does not change significantly. The voltage RMS is calculated by numerically integrating the real-time value of measured voltage in one cycle, and then the power absorbed by the resistor is obtained, as shown in Figure 15b. From Figure 16, it can be seen that the output power reaches maximum when the matching capacitance and load resistance are 13 μF and 13 Ω, respectively, and also the harvester should work under the fundamental frequency of 75 Hz. In the experiment, the maximum output power RMS are 6.7 mW (at 3 g acceleration), 7.5 mW (at 4 g acceleration), 15 mW (at 6 g acceleration), respectively. It can be concluded that the iron-gallium alloy MSM vibration energy harvesting system has the strongest ability to output electric power when the connected load resistance is close to the resistance of the pick-up coils and with a proper matching capacitance. This conclusion also partly described in [36]. Therefore, it is necessary to ensure that the external load matches with the pick-up coils impedance as much as possible. Unlike a piezoelectric energy harvesting system that requires a large matching impedance [23], for example, a corresponding resistance of the ME transducers is 2.4 MΩ [51], a single piezoelectric stack based bidirectional energy harvester is 12 KΩ [52], so an iron-gallium alloy-based harvester requires a smaller matching impedance, which is suitable for driving lower impedance loads. This is because a MSM harvester has inductive properties, not the capacitive properties of piezoelectric harvesters.

**Figure 17:**
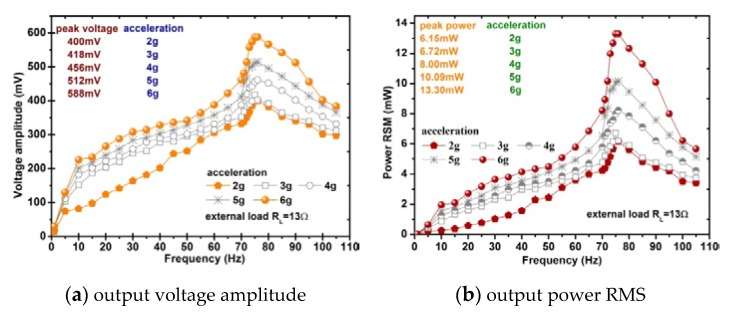
Testing of the voltage and power from the harvester output to the optimal load.

Figure 17 shows the voltage and power from the energy harvesting prototype output to the optimal load. The measured maximum voltage is about 588 mV, output power reaches to 13.3mW, the power density per acceleration (power divided by the total volume of the iron-gallium active layers and acceleration) is 3.7 mW/cm^3^/g which is 19.9 times the power density of the Metglas 2605SA1-based MSM harvester 186 μW/cm^3^/g (the power density is 279 μW/cm^3^ at 1.5 g acceleration) designed by the Hu and Xu [37] and 8.4 times the magnetostrictive/piezoelectric composite harvester 0.44mW/cm^3^/g (the power density is 1.1mW/cm^3^ at 2.5 g acceleration) designed by Dai and Wen [53].

This is the maximum power supply capacity of the preliminary optimized harvesting device working in the range of 2–6 g vibration. The key parameters and performance of the optimized harvester are listed in Table 1.

## 5. Power for LEDs from Harvesting Ambient Vibration Energy

### 5.1. Signal Processing and Energy Harvesting Circuit

It can be seen from the above experiments that the electric energy generated by the vibration harvester is usually an AC with the magnitude of several tens of mV to 2 V. Therefore, it is necessary to design an efficient energy harvesting converter, which not only can convert AC to DC, store the energy, but also track the maximum power so as to use such low power to boost such low AC voltage for satisfying the requirements of the load of electronic device. The energy harvesting converter should have two operating capabilities, first rectification for converting AC to DC, and then DC-DC boosting.

**Figure 18:**
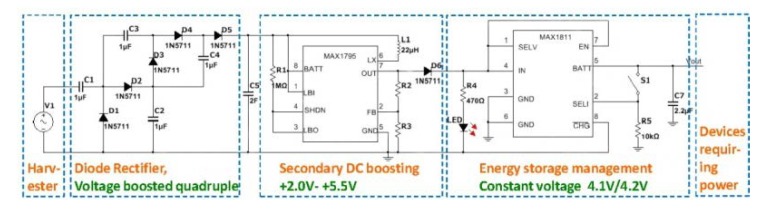
Circuit diagram of the converter based on a quadruple voltage rectification circuit.

Figure 18 shows a converter that includes three parts: diode bridge rectification, secondary DC boosting, and energy storage management circuits. It is a quadruple voltage rectification circuit that can rectify an AC signal into a DC signal and amplify the voltage four times theoretically. An intelligent voltage regulation circuit used as secondary voltage boosting and regulating is designed using a MAX1795 chip. The electrical energy after rectification is stored in the super capacitor C5 temporarily. When the voltage reaches the starting level, the MAX1795 starts to operate normally to perform secondary boost regulation. The output voltage value can be adjusted by the resistor divider principle, and the output voltage is in the range +2.0 V–+5.5 V. In addition, a lithium battery energy storage management circuit is designed using a MAX1811 chip, which provides a constant voltage (4.1 V/4.2 V) or constant current for the final storage of electrical energy.

**Figure 19:**
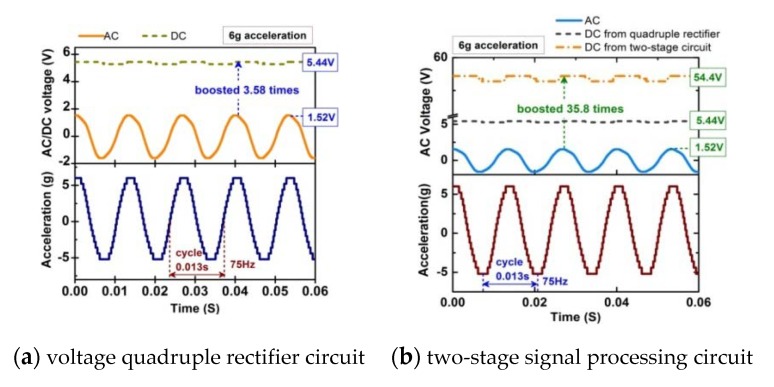
AC output from the harvester and DC output from the energy harvesting converters.

**Figure 20:**
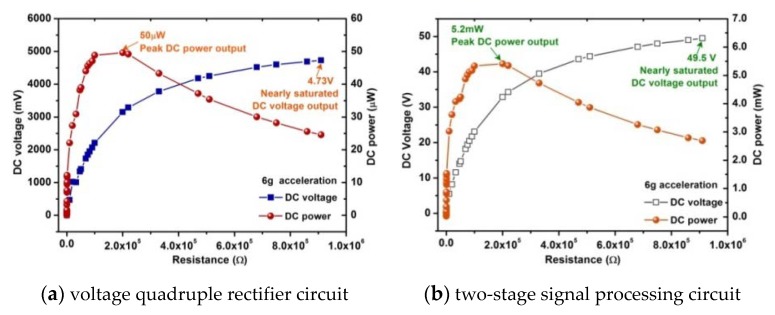
Measured DC output voltage and output power from the converters.

Figure 19a shows the experimental waveforms of the original AC voltage, the amplified AC voltage, the DC voltage from the quadruple voltage rectification circuit, and acceleration of the cantilever beam. In this experiment, the steady state DC output from the quadruple voltage rectification circuit is 5.44 V instead of the theoretical value 6.08 V (1.52V × 4). It is noted that, there is a 0.64 V voltage loss. The conversion efficiency of the converter based on quadruple voltage rectification circuit is about 85% when the external resistance is 20 Ω. One of the reasons for this large voltage drop is the forward voltage drop of diode d1n5711. Second, the operating condition of the entire vibration harvesting system is different from a simple open-circuit condition. It is observed in the experiment that the quadruple voltage rectification circuit continues to conduct current even after the output voltage has reached the steady state due to leakage losses. Any current conduction in the quadruple voltage rectification circuit affects the induced voltage in the pick-up coils [37]. The maximum DC output power of the converter driving a resistive load is 50 μW as shown in Figure 20a. The DC output power density after the converter is 13.9 μW/cm^3^/g.

The energy loss caused by the forward voltage drop of the diode is usually 0.41–1.00 V, for example, the voltage drop of the diode in the above circuit (shown in Figure 18) is 0.64 V, and accordingly the output voltage below 0.10–0.25 V of the energy harvester will be blocked by the rectifier diode, which is characterized by low voltage and low power. For low-voltage, low-power harvester, this means a large part of the available energy. Therefore, a two-stage signal processing circuit is designed, in which a transformer (1:10) is connected between the harvester prototype and the above quadruple voltage rectification circuit to increase the amplitude of the output voltage to a greater extent firstly. And then, the boosted AC voltage is rectified by the quadruple voltage rectification circuit. In theory, the converter can amplify the voltage 40 times, which is much larger than other voltage boosting methods previously proposed. From the result shown in Figure 19b, it can be seen that the steady state DC output from the two-stage signal processing circuit is 54.4 V at 6g acceleration which are about 10 times than that of voltage quadruple rectifier. The two-stage signal processing circuit not only rectifies the AC output to DC, but also the voltage is increased more than 30 times. The conversion efficiency is about 67.2%. Although conversion efficiency is lower than that of the voltage quadruple rectifier circuit mentioned above, the DC voltage after rectification is increased by about 10 times which is because there is no forward voltage drop in the transformer. The reduction of transmission efficiency and power is due to the copper loss and iron loss in the transformer. The maximum DC output power of the two-stage signal processing circuit driving a resistive load is 5.2 mW as shown in Figure 20b. The DC output power density after the two-stage signal processing circuit is up to 1.45 mW/cm^3^/g.

### 5.2. Power Supply Experiment for Electronic Components

**Figure 21:**
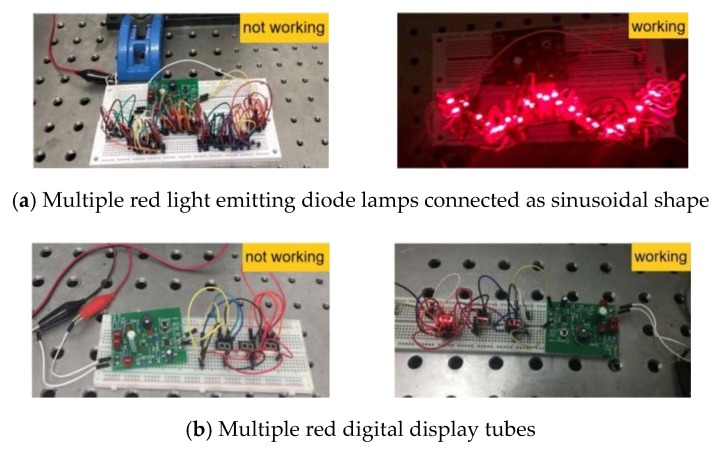
Photographs of a setup in which the prototype is used to light up multiple light emitting diode lamps and multi red digital display tubes.

Finally, we try to use the vibration energy harvesting prototype connected with a converter circuit to power the parallel multiple light emitting diode lamps and multiple digital display tubes. The experimental result is shown in Figure 21. From the experimental photographs, it is noted that the multiple red light emitting diode lamps connected as a sinusoidal shape are lighted up by the prototype, which is shown in Figure 21a. Furthermore, we manage to power multi red digital display tubes using the prototype, which is shown in Figure 21b. This may be the first time a iron-gallium alloy-based cantilever harvester has been used to light up light emitting diode lamps and digital display tubes continuously. This experiment verifies the vibration harvesting capability and electricity-generating capability of the harvester prototype and working effectiveness of the signal processing and energy converter. It allows us image that the iron-gallium alloy based cantilever vibration energy harvester can be used in light emitting diode indicating devices, smart phones, smart bracelets, light emitting diode portable lights, wireless sensor network nodes and so on.

Up to now, all the above experimental analysis can outline the design principles of the cantilever iron-gallium alloy vibration energy harvester. This harvesting device after optimization according to the methods herein has excellent and encouraging performance in terms of design simplicity, output voltage and power and conversion power. At the same time, the influences of some geometric parameters and physical parameters of the system on the overall performance are also qualitatively summarized through experiments. However, as mentioned above, precise system modeling issues considering mechanical structure is necessary to be studied in the future work.

## 6. Conclusions

In this paper, we systematically studied the basic issues related to the design of iron-gallium alloy cantilever vibration harvesters and clarified the impact of these key issues on the energy harvesting and conversion capabilities, including vibration composite beam configuration, and layout of the bias magnets, matching impedance, etc.:

(1) The vibration harvesting performance had obvious dependence on the configuration of the vibration composite beam, and the configuration with multiple active layers was more suitable for obtaining higher energy harvesting and conversion capacity. Moreover, there is an optimal number of active layers to maximize output voltage and power, which is a fairly new conclusion and issue for the design of MSM iron-gallium alloy cantilever harvester.
(2) When the magnet was completely symmetrically distributed above and below the cantilever beam (layout B6), the iron-gallium layer sensed a most suitable bias magnetic field, which had the strongest promoting effect on the energy conversion capacity. The maximum voltage reached to 1.52 V which might be more than 16 times higher than that of without bias magnetic field.
(3) The matching impedance was smaller and an additional high impedance matching circuit was not needed. However, it should be noted that the matching impedance should be considered in the complex domain.
(4) The optimized harvester prototype was capable of supplying the optimal load with 13.3 mW power (RMS) and 3.7 mW/cm^3^/g power density (RMS).

Using the prototype connected with the interface circuit, it successfully powered for multiple red light emitting diode lamps connected as sinusoidal shape and multiple red digital display tubes, which verified the vibration harvesting capability and electricity-generating capability of the harvester prototype and the effectiveness of the signal converter. This is a quite encouraging result that would allow to imagine such harvester to really supply light emitting diode indicators, smart phone, smart bracelet, light emitting diode portable light, wireless sensor network nodes and so on.

## Figures and Tables

**Table 1:** Summary of the key structural and physical parameters and power output of the harvester designed in this paper.

**Pick Up Coils**	**Total Volume (cm^3^)**	**Total Weight (Kg)**	**Configuration of Multi-Beams**	**Muli-Beam Dimensions (mm)**
800 turns, φ 0.203 mm (AWG32), 12.2 Ω, 5.8 mH	1.647	0.014	MB2	Iron-gallium layer: 40 × 15 × 0.5 Elastic layer: 50 × 15 × 0.5
**Fundamental frequency (Hz)**	**Optimal resistance load (Ω)**	**Voltage at the resistor (mV)**	**Power (mW)**	**Power density relative to active materials (mW/cm^3^)**
75	13	6 g acceleration		
		588	13.3	22.2
		4 g acceleration		
		456	8.0	13.3
